# Thinking about hallucinations: why philosophy matters

**DOI:** 10.1080/13546805.2021.2007067

**Published:** 2021-12-07

**Authors:** Sam Wilkinson, Huw Green, Stephanie Hare, Joseph Houlders, Clara Humpston, Benjamin Alderson-Day

**Affiliations:** a Sociology, Philosophy and Anthropology, University of Exeter, Exeter, UK; b Neuropsychology, Cambridge University Hospitals NHS Foundation Trust, Cambridge, UK; c Neuroimaging, University of Maryland School of Medicine, Baltimore, MD, USA; d Philosophy, University of Birmingham, Birmingham, UK; e School of Psychology, University of Birmingham Institute for Mental Health, Birmingham, UK; f Psychology, Durham University, Durham, UK

**Keywords:** Hallucinations, psychosis, philosophy, phenomenology

## Abstract

**Introduction:** Hallucinations research is increasingly incorporating philosophy or the work of philosophically trained individuals. We present three different ways in which this is successfully implemented to the enhancement of knowledge and understanding of hallucinations and related phenomena.

**Method:** We review contributions from phenomenology, philosophy of cognitive science, and philosophy of science and psychiatry.

**Results:** We demonstrate that these areas of philosophy make significant contributions to hallucinations research. Phenomenology gives us a sophisticated and critical understanding of the lived experience of hallucinations. Philosophy of cognitive science enables big-picture theorising and synthesis of ideas, as well as a critical engagement with new paradigms. Philosophy of science and psychiatry raises valuable and theoretically informed questions about diagnosis and categorisation.

**Conclusions:** These contributions reflect both the methodological variety within philosophy and its relevance to the hallucinations researcher.

## Introduction

Hallucinations are typically taken to refer to perceptual experiences that lack a sensory stimulus (e.g., David, 2004). They are a prominent feature of several psychiatric conditions and occur in a minority of the healthy population. A recent growth in hallucination research – in clinical and non-clinical contexts – has been matched by a renewed interest in the theoretical questions that hallucinations pose for philosophical inquiry, encompassing phenomenology, philosophy of mind, and philosophy of science. Correspondingly, insights from theoretical work on hallucinations have informed empirical research and have been conducted in close dialogue with new findings. Here we review how various issues, theories, and models of hallucination relate to contemporary work in philosophy, and what implications this work has for hallucinations research. It will offer a guide for cognitive researchers who are new to philosophical theories and concepts, alongside critiques of dominant contemporary models. Our aim is to show that hallucinations research is intrinsically philosophical, with many of the key questions in this area demanding dialogue and engagement with philosophy.

Hallucination – and psychosis more broadly – has been of particular interest to philosophers for a range of reasons. In the abstract, they can provide the material for various thought experiments and other tests of philosophical theories of perception, belief, and knowledge. This kind of work has been matched recently with the emergence of philosophers of cognitive science – such as Andy Clark, Jakob Hohwy, and Peter Langland-Hassan – collecting empirical data on hallucinations and related topics. The latter approach has often included a focus on how hallucinatory phenomena work in practice and are experienced in day-to-day life. In contrast, the former approach engages much less frequently with hallucinations “in the wild”, and is largely focused on the specific epistemological and ontological questions posed by the possibility of hallucination (see Macpherson & Platchias, 2013). Here we will not focus on this tradition with analytic philosophy, turning instead to how philosophical ideas interface with the real-world experience of hallucination via phenomenology (Section 1), philosophically-informed cognitive models of hallucination (Section 2), and the clinical and diagnostic classification of hallucination (Section 3).

This demonstrates not only the value, but also the variety, of things that we might call “philosophy”. The contribution of philosophy can be in paying close attention to experience (as in Phenomenology), offering conceptual rigour (as in assessment of cognitive models), and offering “big picture” theorising to contextualise contemporary models (as in philosophy of science). Crucially, these are important skills for philosophers and non-philosophers alike in the study of hallucinations, and they can be seen both via the direct contribution of philosophers, and in the empirical practice of cognitive and clinical researchers.

## Phenomenology

The first question we might ask about hallucinations is: what is the experience *like*? To develop answers to this, we can draw upon insights from the sub-field of philosophy known as Phenomenology. “Big-P” Phenomenology, as a philosophical movement, is distinguished from “small-p” phenomenology, which refers to the description of qualitative aspects of experience, in psychiatry and beyond. In what follows we first discuss what Phenomenology is and how it differs from other approaches to describing subjective experience (such as those often employed in clinical practice). We then offer some examples of how Phenomenology has informed hallucinations research in the past – specifically with regards to schizophrenia – and provide a brief survey of contemporary research.

Phenomenology as a movement took full form in the early twentieth century. Edmund Husserl is typically credited as the founder of the field, owing to the publication of his influential work *Logical Investigations* (Husserl, 1900/2001).^1^ There is an on-going debate about exactly what Phenomenology is and does: for example, the Phenomenology of Husserl, Heidegger and Merleau-Ponty, differ in their understanding of what Phenomenology *is*, and therefore what it *is for*. However, two relatively invariant features of Phenomenological work are (1) its emphasis on rich description of first-person experience, in particular what are known as “structures of experience”; and (2) its aim to be “atheoretical”, i.e., to *not* set out with a particular theory about human experience that is proved or disproved – only try to describe what it is typically like. Broome et al (2012, p. 1) describe these two features in the following way: “Phenomenology emphasises description or elucidation rather than explanation or analysis. […] Phenomenology works to avoid “blinkers” and prejudice: perhaps the strongest appeal to empirically minded psychiatrists lies in this idea of avoiding theoretical assumptions and distortions”.

Some further discussion of (1) is required here to distinguish it from mere rich description of subjectivity. Examples of structures of experience include “temporality”, “embodiment”, “intentionality”, and “selfhood” (including “ipseity”, or a basic experience of self).^2^ These are interrelated aspects of experience that, whilst given to all kinds of alteration, are always present in one form or another. For example, regardless of what we are experiencing, it will include to a greater or lesser degree an experience of *time*: watching a leaf falling, reaching for a cup of coffee, travelling abroad, aging, will all comprise experiences of time. Also present in these experiences is our embodiment, for example, how we are sensitised to possibilities for action – the possibility of catching the falling leaf. And the experience will include a basic sense of “for-me-ness”, a minimal awareness of oneself as the locus of experience. Owing to its specific focus on structures of experience, the approach of Phenomenology is more than describing subjective experience in detail – in the way that, for example, a poet or a clinician who is unfamiliar with Phenomenology might. This emphasis on structures of experience marks Phenomenology as a unique tool in understanding first-person experience, including first-person experience of hallucinations.

Phenomenology (with a little p) is sometimes used as a technical term in clinical disciplines to refer to qualitative signs and symptoms of an illness: “Within Anglo-Saxon psychiatry, the term phenomenology appears to primarily refer to the description of externally observable symptoms” (Burgy, 2008, p. 1206); see also Beumont (1992). Little-p phenomenology is distinct from Phenomenology in that it does not by definition include, or develop from, analyses of structures of experience. In some countries – such as the UK or USA – psychiatric training is arguably not strongly informed by Phenomenology, while others, such as Italy do involve a greater phenomenological emphasis (Fiorillo & Ventriglio, 2018).

The above notwithstanding, there is a rich tradition of using ideas from Phenomenology to inform psychiatric thought and practice. The philosopher-psychiatrist Karl Jaspers is typically credited with first bringing these disciplines into dialogue (Jaspers, 1968, 1997; Stanghellini & Fuchs, 2013). Drawing from this field, our discussion focuses on Phenomenological analyses of hallucination *in the context of mental illness* – as opposed to accounts of hallucinations which occur in other contexts, for example, side-effects of medicine; intended effects of psychedelics; or in nonclinical populations with no need for care. This emphasis does not reflect a general judgment made by the authors about the cause or status of hallucinations in general – the experience of hallucinations is evidently not restricted to the types of suffering typically referred to as “mental illness”. Our emphasis is, however, partially a reflection of how influential analyses of hallucination in the field of Phenomenological psychopathology have proven to be.

The natural place to begin when discussing phenomenological approaches to hallucinations is the “Early Heidelberg School of Psychiatry”. Key members of the School used insights from Phenomenology to develop psychiatric theory and practice, and in particular to “overcome the initial tendency to merely verbally assert a theoretical “essence” of schizophrenia, which putatively “explains” all its symptoms, without basis in replicable method” (Mishara et al., 2014). Members of the school offered a number of interpretations of experiences associated with schizophrenia, including hallucinations, which implicated subjectively experienced disturbances in selfhood (“Ichstörungen”; Kaminski et al., 2019; Sterzer et al., 2016). This work was strongly informed by Phenomenological work on the basic structure of experience – for-me-ness or mine-ness – that was seemingly altered (or perhaps missing altogether) in some psychopathological experiences. For example, Wilhelm Mayer-Gross proposed that hallucinations associated with schizophrenia occur due to disrupted “low-level perceptual processing” of predominantly spatio-temporal information. This disruption impacts upon one’s basic sense of self, leading in turn to misattribution of thoughts as being implanted or somehow externally caused. While Phenomenology can sometimes appear overly abstract and conceptual, examples of it have led to testable hypotheses in cognitive neuroscience (e.g., Giersch and Mishara (2017)).

In a more contemporary Phenomenological approach, Louis Sass (2003) has provided a “two-faceted disturbance of self-experience” account of schizophrenia, with implications for the understanding of hallucinations. The two facets are (i) *hyper­-reflexivity* in which aspects of self-experience that are normally taken for granted become objects of awareness, and (ii) diminished *self-affection*: “a profound weakening of the sense of existing as a subject of awareness, as a presence for oneself and before the world” (Ibid, p. 242). Sass suggests that both facets reflect alterations in a basic experience of self (e.g., ipseity, or self-disturbance). In developing his thoughts he also draws from Merleau-Ponty’s notion of the intentional arc (Merleau-Ponty, 1945/2012, p. 137).^3^ Based on this understanding of schizophrenia, Sass interprets auditory verbal hallucinations (AVH) as involving “exaggerated self-consciousness, that is, a sense of alienation from and a bringing-to-explicit-awareness of the processes of consciousness itself” (Ibid, p. 254). Again, attentiveness to subtle variations in self-experience, drawing on a range of Phenomenological insights, has added weight to the view that subtle disturbances in self-experience are relevant to schizophrenia, in particular the prodromal phase – these then lead to more frank symptoms such as hallucination. A helpful review of how Phenomenological analyses, including Sass’s, have informed this hypothesis is provided by Nelson et al. (2009).

There is also considerable *breadth* across contemporary phenomenological approaches to hallucination, typically by applying ideas and concepts from “big P” into “little p” research. Pienkos et al. (2019) discuss expanding existing definitions of hallucination, based on Phenomenological analysis of experiential alterations associated with psychosis (such as alteration in a person's sense of wider reality). Elaborating on existing definitions of hallucination would naturally have implications for future research. Woods et al. (2014) make a compelling case for how Phenomenological research, in tandem with other disciplines, can “nourish the ethical core of scientific enquiry by challenging its interpretive paradigms, and offer voice hearers richer, potentially more empowering ways to make sense of their experiences” (Woods et al., 2014, p. 246). Here Phenomenological insights are presented as a possible heuristic for voice-hearers to interpret their experience, and could be further speculated on as having some form of *therapeutic* value (i.e., improved understanding perhaps making experiences less mysterious or frightening). In a comparative phenomenological approach, Luhrmann et al provide an analysis of potential links between trauma and experiences of AVH, proposing that there are “seemingly distinct phenomenological patterns for voice-hearing, which may reflect the different salience of trauma for those who hear voices” (Luhrmann et al., 2019, p. 24). Humpston and Broome (2015) use Phenomenology to question the traditional distinction between AVH on the one hand and thought insertion on the other, proposing that there is a spectrum ranging from loud thoughts, all the way to audible voices. Last, Ratcliffe (2017) has emphasised the importance of relationships with other people in the experience of this form of hallucination, in a thorough examination of AVH phenomenology. Ratcliffe draws on various Phenomenological insights in developing his claim, including Husserl’s discussion of certainty and the role that other people have in the formation of this (Ibid, pp 140–190). These five examples testify to the diverse range of ways Phenomenological research is presently informing understanding and research into hallucinations.

The discussion above exemplifies our view that adopting a Phenomenological perspective is conducive to maintaining an openness to the complexity of human experience, including hallucinatory experiences. Furthermore, existing Phenomenological work on structures of experience provide a blueprint for exploring different experiential dimensions of hallucination. In this way Phenomenology can function as a “methodological mirror”, i.e., reflecting the nuances of the object of study (for example, patient experience). This section has sought to demonstrate the unique offering of Phenomenology to hallucination research, and to distinguish it from mere rich description of first-person experience. Phenomenology is well equipped to elucidate the often subtle heterogeneity of hallucinations, and to develop a detailed picture of *what it is like* to have particular kinds of hallucinatory experience.

## Philosophically informed cognitive models of hallucination

Most cognitive researchers of hallucination will be concerned less with the immediate phenomenology of the experience, and instead seek to explain *how* they occur as a result of psychological and neurobiological processes. There have been a range of causal models proposed in relation to hallucinations, largely based on those experienced during psychotic states and, in particular, schizophrenia. Here we focus on two theories which have shaped approaches to hallucination: the self-monitoring model – in which hallucinatory states are cast as internal mental events, misattributed to another – and the more recent predictive processing approach, where self-generated models of the world determine perceptual experience.^4^ Although these are both theories that have been developed within cognitive and computational frameworks, their adoption, development, and impact have all involved contributions from philosophy.

The origins of self-monitoring are in theories of motor control. Typically attributed to theorists such as Irwin Feinberg (1978) and Chris Frith (1992), the basic premise of this approach to psychotic states is that motor actions are accompanied by monitoring processes, in which an action and its expected sensory outcome are compared. Developed originally as a model for passivity experiences and delusions of control, Frith (1992) proposed that people with schizophrenia might be impaired in self-monitoring, leading to self-generated actions being experienced as controlled by another. The extension to hallucinations – and in particular AVH – was to propose that thoughts (or inner speech) could be generated and misattributed via a similar process, leading to the perception of hearing voices (e.g., Jones & Fernyhough, 2007; Seal et al., 2004).

The predictive processing approach, in contrast, largely grew out of computational approaches to vision (Rao & Ballard, 1999) and the work of figures such as Karl Friston (2005) and Steven Grossberg (2000). It has now been applied to many aspects of action, perception and cognition. Predictive processing approaches to hallucination take the central idea of predictive coding – that all perception is a probabilistic generative model of the world, corrected only when necessary by sufficient prediction error signal – and emphasise imbalance in this process for people with hallucinations, in which predictions based on prior evidence (referred to as “priors”) are given too much weight or not enough weight. Perception can then become biased in various ways – for instance, to hear voices.

Philosophy has contributed to the transition between these two approaches, both in critique of the self-monitoring approach, and in the enthusiastic interrogation and application of predictive processing ideas. Philosophers are particularly adept at recognising (sometimes hidden) key assumptions of a particular theory. A key assumption of the self-monitoring theory, with important philosophical implications, is that verbal thought is cast as a form of motor action, with corresponding processes of planning, monitoring, and sensory prediction. Working from a phenomenological perspective, Gallagher (2004) for example has critiqued the ability of self-monitoring models to explain experiences such as inserted thoughts, relevant here given their similarity to AVH. This highlighted such problems as specificity (why are only some thoughts misattributed?) and the apparent experience of not less but *more* monitoring of one’s thoughts that is often described in psychosis (the experience of “hyper-reflexivity”). This critique in itself built on another response to the self-monitoring model, put forward by Stephens & Graham (2000), that Frith did not fully explain why a failure to experience an action or thought as one’s own necessarily entails the attribution to –or perception of– another agent. Similar arguments have been taken up recently by philosophers and psychologists emphasising the irreducible social and agentic nature of hallucinations in particular and psychosis in general (Bell, 2013; Wilkinson & Bell, 2016).

Along with the above concerns, the self-monitoring model became invariably tied to misattributed inner speech production as a model for AVH (hallucinations in other modalities – such as smell and taste – being possible to account for, but not convincingly). Predictive processing, in contrast, has no such tie to a specific modality, and no tie to motor processes – being a model posited for all perception. And rather than being a rejection of the self-monitoring idea, in many cases predictive approaches have often been cast as necessary extensions of the concept, or at least conciliatory with it (Corlett et al., 2019; Fletcher & Frith, 2009; Griffin & Fletcher, 2017; Pickering & Clark, 2014; Wilkinson, 2014). This highlights another critical advantage of philosophy in recognising that models of hallucinations are not necessarily mutually exclusive and to interrogate deeper the shared features of theoretical approaches. This can counter a tendency in cognitive research for researchers to work within their given theoretical silo, treating their theoretical perspective as wholly unique and superior, when, in fact, there is great overlap in the core assumptions of many neurocognitive models.

Philosophical analysis may also help highlight important trade-offs between dominant theories of hallucinations. For instance, the emphasis on speech-motor processes as particularly prone to disruption in self-monitoring theory would appear to fit well with the preponderance of AVH in psychosis compared to other modalities (typically around 75% of people with schizophrenia experience AVH, compared to less than 50% for other modalities; Bauer et al., 2011). Predictive processing, in contrast, provides no direct account of why some modalities may incur hallucinations more than others. Conversely, however, predictive processing, in making prediction central to both self-produced and exogenous experiences, can account for “passive” symptoms (Wilkinson, 2015) that have no clear motoric elements, such as “hypervigilance hallucinations” (Dodgson & Gordon, 2009).

Nevertheless, the extreme flexibility of the predictive processing approach has entailed theoretical strengths and weaknesses which philosophers have been influential in emphasising and elucidating. Work by Clark (2013, 2015) and Hohwy (2013) has done much to emphasise the broader implications of adopting predictive processing as a model of the mind, including its relations to imagination and social cognition. At the same time, the theory’s scope has led to the accusation that they act as a “catch-all” theory that is potentially consistent with everything, and hence difficult to falsify (see Williams, 2018). In contrast to the empirical sciences, philosophers have arguably been the most prominent critics of the predictive approach (e.g., Colombo et al., 2020), alongside the advocacy provided by Clark, Hohwy and others.

One of us (Wilkinson) has argued that predictive processing offers a new kind of description of a range of phenomena, that is helpful for some purposes, rather than a complete explanatory account of why they occur (Wilkinson, 2014). These kinds of trade-offs will be familiar to researchers with training in philosophy of science, given that the virtues of each approach differ in quite contrasting ways (e.g., breadth, depth, parsimony, etc.), and serve to limit their explanatory appeal. The effect of this is arguably a much longer “transition period” than scientific theories would ideally inhabit. Accordingly, the self-monitoring model has not gone away, but has been extended via further exploration of the varieties of inner speech and mental imagery. Previous philosophical approaches to inner speech have often assumed a certain uniformity of experience and function (e.g., Carruthers, 2002), but a new wave of philosophers have sought to empirically examine the phenomenology, diversity and functions of inner speech, including Peter Langland-Hassan (Langland-Hassan et al., 2015), Valentina Petrolini, Agustin Vicente, and Marta Jorba (Petrolini et al., 2020).

Looking ahead, contemporary philosophical approaches to hallucination may be particularly valuable in helping us to understand their relation to memory, trauma, and affect. Some hallucinations (in psychosis, and particularly in post-traumatic stress disorder (see Wilkinson et al. 2017)) are closely related to prior experiences, and may even be experienced as “replays” of earlier events. Memory models of hallucination have typically drawn upon the clinical concept of dissociation, whereby memory is encoded in a fragmentary way during traumatic experiences, and the self-experience can be shut off and inaccessible to the individual. How and why particular memories may become experienced as external *percepts*, however, is not always fully elucidated in such models (e.g., Longden et al., 2012).

In a related manner, researchers working on predictive processing have moved from work on affect and emotion in general (Barrett, 2017 Seth, 2013;), towards work on affective *valence* (Deane et al., 2020; Van de Cruys 2017) (i.e., positive vs. negative valence). Although emotion and affect are often treated synonymously, within predictive processing different accounts emerge. Recently, Deane et al. (2020) have tied affect to control (in the low-level sense that your nervous system is doing a good job of minimising prediction error), and, in turn, tied this to notions of the self as a way of understanding positively and negatively valenced self-less experiences in the contexts of meditation and depersonalisation respectively. A potentially exciting future direction might be to examine how this notion of control fits with valence in AVHs, not only in the generation of an aetiological account, but also in terms of predicting correlations between control and valence of AVH experiences (i.e., low levels of control would be negatively valenced). There may also be a connection between this research and the phenomenological research on altered self-experience and psychosis (Sass & Parnas, 2003).

## Diagnostic classification

Philosophers of science in general, and of psychiatry in particular, have examined the role of scientific classifications in shaping the manifestation and understanding of psychiatric phenomena. This has relevance for psychopathology, where an enduring difficulty has been the question of what (disorders, symptoms, experiences) constitutes a valid object of study.

Recent hallucination research has been strongly influenced by the idea that symptoms are a more valid object of study than putative disease categories (i.e., the “post Kraeplinian psychiatry” proposed by Bentall: “Research into psychological complaints must start from a detailed description of those complaints” Bentall, 2003, p. 143). On this view, “hallucinations” can be understood as Lego blocks serving as constitutive elements of more complex kinds (e.g., diagnosis of schizophrenia, diagnosis of Parkinson’s disease, psychic experiences, etc.).

The “post Kraeplinian” focus on symptoms arises out of the position that diagnoses are scientifically meaningless and conceptually confused (Bentall, 2003; Boyle, 2002). It is proposed that we should instead research symptoms (or “complaints” for those who have found the terminology of “symptom” and “illness” inappropriate). Even absent the explicit endorsement of the strong position that diagnoses are scientifically meaningless, there seems to be an implicit assumption that researchers should focus on delineating the mechanisms of more *basic* phenomenological and behavioural constructs such as hallucinations.

The Lego-block view of disorders raises a question: If diagnoses are not natural kinds (i.e., real things that we find in the world), perhaps symptoms might be? An essential feature of natural kinds is our ability to draw inferences from them. In the case of gold we can always expect a reaction between gold and element Y to produce compound Z. However, the diversity and unpredictability of human psychology and behaviour makes it difficult to glean straightforward inferences (e.g., X will *always* produce Y).

Considered as a natural kind, “schizophrenia” falls short. The recent history of the diagnosis has seen new neurological illnesses cleaved away from it (e.g., NMDA-r encephalitis; or syndromes arising from rare genetic variations – see Mitchell, 2019, for a discussion), suggesting it is in fact a broad syndrome that likely represents multiple different phenomena. Nonetheless, we can make some probabilistic inferences on the basis of the category: If person A has a diagnosis of schizophrenia, then they (a) are likely to have some degree of functional impairment; (b) are likely to have other symptoms such as delusions, disordered thought or cognitive impairment; and (c) are more likely to be male than female.

“Hearing voices” or AVH appears intuitively a more promising candidate as a psychological phenomenon that could be indexed to a physiological natural kind. Not only in virtue of its superficially more basic character as a constitutive element of a broader diagnostic category, but also for its appeal (in the former case) to ordinary language. There are reasons to suppose hallucination is a variable more tightly linked to candidate cognitive endophenotypes than diagnostic categories such as schizophrenia. For example, auditory hallucinations are associated with intentional cognitive inhibition, a specific cognitive mechanism (Alderson-Day et al., 2019) while schizophrenia is associated only with much broader and less specific inhibitory tasks. There is also evidence that the visual hallucinations of Lewy Body Dementia (Zarkali et al., 2019) are associated with the same pattern of overweighted priors, on the same tasks, as in psychosis (Teufel et al., 2015), and in induced hallucinations in healthy volunteers (Powers 3rd et al., 2017). Additionally, there are neurological differences between voice-hearers and non-voice hearers, regardless of diagnosis (Powers et al., 2020)

Nonetheless, although hallucination is a more scientifically specific phenomenon than schizophrenia, it is not clear– for practical purposes – that it will always be the most salient level of analysis for understanding a person’s experience. It is sometimes suggested that hallucinations in the context of a functional psychotic illness are different in kind from hallucinations in the context of neurological disease. Oliver Sacks reflects this clinical commonplace when he says “the hallucinations often experienced by people with schizophrenia also demand a separate consideration, a book of their own, for they cannot be divorced from the often profoundly altered inner life circumstance of those with schizophrenia” (Sacks 2012, pp xiii). Sacks’ idea bears further scrutiny – what psychopathological facts might bear on this apparent distinction?

The suggestion is that a functional psychotic disorder like schizophrenia is not just another context in which hallucinations appear, rather a schizophreniform hallucination is potentially different in kind than a hallucination in the context of a neurodegenerative disease. Hallucinations in neurological disorders are often felt to be transient and well-formed, with patients readily accepting their unreality (Groh-Bordin & Kerkhoff, 2010). However, hallucinations in psychosis are often more pervasive, frightening and woven into the worldview and belief system of the people who experience them. The particular *qualities* of a hallucination (explored in detail in the work of some phenomenologists – such as Merleau-Ponty) are part of what determine whether it is schizophreniform.

Philosophically inclined psychopathologists have argued that difficulties with the DSM construct of schizophrenia do not necessarily entail incoherence in a broader disorder concept. For example, Kendler (2017) argues that the DSM criteria are indexical *for* and not constitutive *of* psychiatric disorders. Gipps (2020) argues that schizophrenia’s thoroughly articulated phenomenological character (see elsewhere in this article) renders the positivist project of defining a schizophrenia construct comparatively unimportant, as compared to the project of elaborating the distinctive character of “schizophrenic” psychoses. This perspective allows that clinicians and researchers are still warranted in distinguishing between a hallucination in the context of a schizophreniform illness, a hallucination in the context of a neurological problem, and a hallucination in a person with no diagnosis. Partly this is because hallucination is a phenomenon that is particularly difficult to divorce from its psychopathological context – from the perspective of the individual. A person who hears voices as well as experiencing paranoid ideas is likely to understand their experience of voice-hearing differently. Equally, different types of hallucination may have different effects on other co-occurring symptoms (vividness of hallucinatory experience might contribute to an increase in anxiety). We suggest that the bidirectional relationships between symptom and disorder context make it impossible to disregard contextual information, such as psychiatric diagnosis.

There are, for example, significant clinical and phenomenological differences between (i) a person who fleetingly sees shadows and images of a figure in a white cloak, who knows that the things she sees are not real and attributes them to the effects of a brain injury she had when she was younger, versus (ii) an individual that hears voices constantly screaming and criticising her, that she takes to be real and that are plotting against her. To what extent are these experiences different, such that we might say that one is an instance of “hallucination” but the other is not? And to what extent is it driven by a broader symptomatic context, such as a more generalised difficulty with reality testing or source monitoring?

These considerations suggest some potential avenues for research. One is a change in how we conceptualise the relationship between symptoms and the disorders to which they are linked. Although there may be fundamental neurological similarities between hallucinations across disorders, the Lego block metaphor is potentially restrictive – a better model might be that of the ingredients in a cake. Symptoms alter the minds in which they appear, but the relationship is likely bidirectional.

Understanding the reasons for these perceived differences is a project that requires the drawing of between-disorder comparisons. For example, Powers 3rd et al. (2017) compared voice hearing in clairaudient psychics to voice hearing in people with psychotic illness; finding that in the former group these experiences were associated with less distress. Luhrmann et al. (2019) have argued that voice-hearing in the context of trauma manifested distinct phenomenological patterns compared with voice-hearing in the context of psychosis. Equally, network analyses of psychotic symptoms and recent life events promise to shed light on the interaction of symptoms in context (Betz et al., 2020).

Another consideration illuminated by philosophy – and with implications for the “Lego block” view – is the historically mutable character of the definition of hallucination. What gets included in the category “hallucination” has varied across time as hallucinatory experiences have been subject to shifting definitions (see, e.g., Berrios, 1996). Additionally, researchers have given more or less focus on different *aspects* of the hallucinatory experience, depending on the cultural and medical priorities of the moment. The result of this variation has been that the category of hallucination has previously included experiences that might now be considered something else, and that some aspects of hallucination – once considered significant – are now forgotten.

Consider the historic distinction between hallucinations that do and do not have a sense of “objective reality”. This was once regarded as an important way to demarcate “hallucinations” – thought to be associated with psychosis and neurological illness – from “pseudo-hallucinations”, experiences that are phenomenologically similar to hallucinations but involved “intact reality testing”, and have an association instead with personality and conversion disorders (van der Zwaard & Polak, 2001). Lately the distinction found more concrete definition in the differentiation between hallucinations that do or do not occur from “outside” a person’s head – but subsequent phenomenological research has demonstrated its failure to track either diagnostic status or severity (e.g., Copolov et al., 2004). The distinction has since been discarded in many cases as clinically irrelevant and is rarely used in contemporary AVH research (Nayani & David, 1996; van der Zwaard & Polak, 2001).

Researchers also make a number of distinctions between different types of psychotic phenomena, when they may obscure important connections. For example, while it is commonplace to distinguish thought insertion from hallucination, some have highlighted the potential connections and interactions between these phenomena, along a spectrum of audible thoughts (e.g., Humpston & Broome, 2015). The most significant ongoing distinction in psychosis is between hallucination and delusion, though even this has been undermined, by suggestions that delusions could be understood as resulting from “cognitive hallucinations”, (an experience of thinking that you believe something which you don’t actually believe) (Currie, 2000) and by the influence of the predictive processing models, which have substantially collapsed the distinction between perception and cognition.

Official definitions and simulations such as those found in DSM-5 not only feed into professional discourse, but also influence media and other cultural representations of the experience of hallucinations. These in turn help shape society’s shared understanding of what sort of thing a hallucination is. The result is that some experiences come to be understood as entailing something serious and/or dangerous (consider the cultural fear that to “hear voices” entails that one is “crazy”), while others are viewed as benign, trivial or possibly overlooked altogether if they don’t resemble canonical definitions. This latter dynamic can lead to experiences of erasure and exclusion. For example, recent work has suggested that some people experiencing psychosis have felt their experiences “lack legitimacy”, (see Jones & Shattell, 2016) as clinicians rely on operational definitions that emphasise experiences that are “vivid and clear, with the full force and impact of normal perceptions” (APA, 2013).

One of us (Green, 2019) has argued that such historical changes in the definition of hallucination are capable of changing the nature of hallucinations themselves, via “looping effects” (Hacking, 1995) and the highly suggestible nature of conscious experience (Schwitzgebel, 2011). The argument runs thus: Our imperfection as introspectors makes us vulnerable to mis-identifying or misdescribing the character of our experiences. Meanwhile, the availability of some popular or canonical descriptions of psychic phenomenology makes it possible for those descriptions to invoked (in research or psychiatric interviews) to crystallise what was in fact an *indeterminate* experience. Experiences are thus misrepresented in research, and potentially even themselves changed. With time, the category hallucination changes too. The way we understand hallucinations is thus influenced by the way we ask about them. This longitudinal and cultural mutability of hallucinations raises questions about the cultural and neurological characteristics of these experiences, potentially opening up a new dimension along which to study them.

Such considerations open up a number of directions for future research. First, we would argue that there is scientific value – not just a hobbyist’s interest – in the historical study of psychopathology. While some distinctions and definitions may have been justifiably discarded as scientifically worthless, others may be simply unfashionable and warrant continued interest. Better understanding the way these symptoms have and haven’t changed over time could contribute to our understanding of the nature of hallucinations. Research frameworks such as NIMH’s RDoC focus primarily on the neurobiological mechanisms underlying symptoms, but there should also be systematic focus on cultural mediators of hallucinatory experience. And, combining these considerations, what neurological mechanisms are implicated in the cultural shaping of hallucination? Are some types of hallucinations – for instance those connected with trauma, or with delusional ideas – more modifiable or changeable than others? Such considerations potentially have value for therapies, shedding light on the role of the cultural milieu for moderating symptom expression and distress.

Finally, we note that those who drive the research agenda are inclined to change their priorities, with more or less focus on different aspects of the hallucinatory experience depending on what is fashionable or likely to be funded. These shifts in priorities in turn shape which researchers have influence, which further shapes research priorities. This sort of cyclic effect is likely to narrow the range of ideas about hallucinations, or at the very least make it much harder to draw meaningful comparisons across different periods and countries. Such research should aim to be inclusive of as broad a range of people as possible; extending to not only research scientists and clinicians, but also service users and other stakeholders.

## Conclusion

We have presented three very different ways in which philosophy can make contributions that further our understanding of hallucinations. This is certainly not an exhaustive presentation. We have discussed how phenomenology can open up research to nuance and variety, and steer us away from oversimplified accounts of the phenomena of interest (in this instance hallucinations, and related experiences in psychosis). Philosophy of cognitive science, with its big-picture theorising and conceptual clarity, can help us to understand sweeping paradigm shifts and reflections on the viability of certain explanatory frameworks and theories. Finally, philosophy of science and psychiatry can cast a critical eye on our practices of scientific categorisation and diagnostic classification.

Empirical researchers, in whichever discipline, using whatever method, quite rightly need to focus on the generation of data and results. But it is also important that philosophically trained individuals, who are also scientifically literate, take the time and space to reflect, collate and critique so as to help steer the research in optimal directions.

## Notes

1 However, Husserl’s thinking was heavily influenced by Franz Brentano’s work on intentionality. Intentionality is a technical term in philosophy to describe the capacity of minds/mental states/consciousness to be *about* other things, e.g. other entities.

2 The term “structures of experience” is a matter of some debate. See Fernandez and Køster (2019) for recent discussion.

3 Hubert Dreyfus describes the intentional arc as a: “feedback loop between the embodied agent and the perceptual world” (Dreyfus, 2014, p. 253)

4 Because of the association with psychosis, both approaches have mostly focused on auditory and, to a lesser degree, visual experiences. Conditions with primarily visual hallucinations (such as Parkinson’s Disease, or dementia with Lewy-Bodies) have generated other cognitive models focusing on visual attention, but these have been much less influential in broader debates about hallucination. As such, they will not be discussed here.
